# Mobile Phone App Aimed at Improving Iron Intake and Bioavailability in Premenopausal Women: A Qualitative Evaluation

**DOI:** 10.2196/mhealth.4300

**Published:** 2015-09-28

**Authors:** Davina Mann, Lynn Riddell, Karen Lim, Linda K Byrne, Caryl Nowson, Manuela Rigo, Ewa A Szymlek-Gay, Alison O Booth

**Affiliations:** ^1^ Centre for Physical Activity and Nutrition Research School of Exercise and Nutrition Deakin University Burwood Australia; ^2^ School of Psychology Faculty of Health Deakin University Burwood Australia

**Keywords:** cell phones, telemedicine, mobile apps, iron, behavior therapy, focus groups, goals

## Abstract

**Background:**

Low iron intake can lead to iron deficiency, which can result in impaired health and iron-deficiency anemia. A mobile phone app, combining successful dietary strategies to increase bioavailable iron with strategies for behavior change, such as goal setting, monitoring, feedback, and resources for knowledge acquisition, was developed with the aim to increase bioavailable iron intake in premenopausal women.

**Objective:**

To evaluate the content, usability, and acceptability of a mobile phone app designed to improve intake of bioavailable dietary iron.

**Methods:**

Women aged 18-50 years with an Android mobile phone were invited to participate. Over a 2-week period women were asked to interact with the app. Following this period, semistructured focus groups with participants were conducted. Focus groups were audio recorded and analyzed via an inductive open-coding method using the qualitative analysis software NVivo 10. Themes were identified and frequency of code occurrence was calculated.

**Results:**

Four focus groups (n=26) were conducted (age range 19-36 years, mean 24.7, SD 5.2). Two themes about the app’s functionality were identified (frequency of occurrence in brackets): interface and design (134) and usability (86). Four themes about the app’s components were identified: goal tracker (121), facts (78), photo diary (40), and games (46). A number of suggestions to improve the interface and design of the app were provided and will inform the ongoing development of the app.

**Conclusions:**

This research indicates that participants are interested in iron and their health and are willing to use an app utilizing behavior change strategies to increase intake of bioavailable iron. The inclusion of information about the link between diet and health, monitoring and tracking of the achievement of dietary goals, and weekly reviews of goals were also seen as valuable components of the app and should be considered in mobile health apps aimed at adult women.

## Introduction

Iron deficiency (ID) is the most prevalent micronutrient deficiency in the world [1]. Insufficient iron can impair oxygen transport due to a reduction in hemoglobin, which in turn can result in impaired mental function, chronic fatigue, impaired aerobic metabolism, decreased work performance, decreased thermoregulation, and decreased immune function [2-4]. ID and iron-deficiency anemia can also negatively affect neuropsychological factors such as mood, cognitive functions, learning ability, and memory [5-8].

Women of reproductive age are at risk of ID due to blood loss via menstruation, low dietary iron intake, and high gestational requirements [9,10]. A few small-scale, resource-heavy, dietary interventions have been shown to increase iron stores in women whose iron stores are low [3,11,12].

Mobile phone apps have potential as a flexible, tailored, customizable, wide-reaching, cost-effective, and accepted means of health promotion [13]. Apps have the ability to incorporate the most effective components of behavior change, such as goals, self-efficacy, self-monitoring, feedback, tailoring, and planning, into a platform that is user friendly, engaging, and flexible [14,15]. Because of the potential of apps to reach a wide audience, we have recently developed an app aimed at increasing women’s intake of bioavailable iron in order to improve iron status. The app has been developed based on dietary interventions that have improved iron status and components of behavior change, which have proven effective in dietary behavior change [3,11,12]. This app will be used in a randomized controlled trial investigating the feasibility of increasing iron intake via supplements or dietary intake using mobile phone apps (ACTRN12613000912785). Pending the outcomes of this trial, the app is intended for wide release and will be made available through various venues such as the iTunes app store and Google Play store.

While apps have the potential to bring dietary and lifestyle interventions to large populations, their efficacy is currently unknown. Prior to the use of this novel app in a randomized controlled trial, usability and functionality of the app should ideally be tested in the target population of premenopausal women. Such information can help to optimize app functionality and effectiveness, to ensure content quality, and to understand the app’s potential for health and behavior change [16-18]. Modifying the development of this app based on initial feedback from potential end users is consistent with recommendations that state a high level of usability is essential to ensure uptake and adoption of health promotion apps [19].

The aim of this study is to evaluate the content, usability, acceptability, and functionality of a mobile phone app designed to improve bioavailable iron intake in premenopausal women. The findings from this study will be used to inform the refinement of the app and add to the evidence base of desired features of apps designed to promote dietary change.

## Methods

### Study Design

This study descriptively explored user preferences for an app designed to improve intake of bioavailable iron in premenopausal women. Participants were provided with a link to download the mobile phone app and were asked to use the app at their leisure over at least a 2-week period, during which time they were asked to read information, play games, set goals, and take photos of meals. Continued use of the app was encouraged via a reminder email sent to each participant during the trial period. Following this 2-week period, semistructured focus group discussions were conducted to determine users’ opinions of the app.

### Mobile Phone App Development

The content of the Women’s Iron, Zinc, and Energy (WIZE) app was developed based on a systematic review of the literature on dietary factors influencing iron status in combination with findings from 2 dietary intervention studies investigating dietary strategies to increase iron status in women [11,20-22]. In addition to providing information on rich sources of dietary iron, the app also included recommendations to increase iron-fortified food intake, consume foods rich in vitamin C with meals, and avoid consumption of tea and coffee with meals, based on previous studies highlighting efficacy [11,20-23]. The app incorporated successful strategies of behavior change such as those found in social cognitive theory [24], the health belief model [25], and self-determination theory [26]. Strategies for behavior change that were utilized by the app included goal setting, monitoring, feedback, self-motivation, and strategies for knowledge acquisition.

The app delivered iron-related nutrition information in 7 fact sheets, which were designed to increase knowledge of dietary sources of iron and the relationship between iron and health. In addition to increasing participant knowledge about iron, it was hoped that informing users about the detrimental health effects of low iron levels would help to make iron status personally important to users. It is theorized that this would promote self-motivation within users and further encourage behavior change.

**Figure 1:**
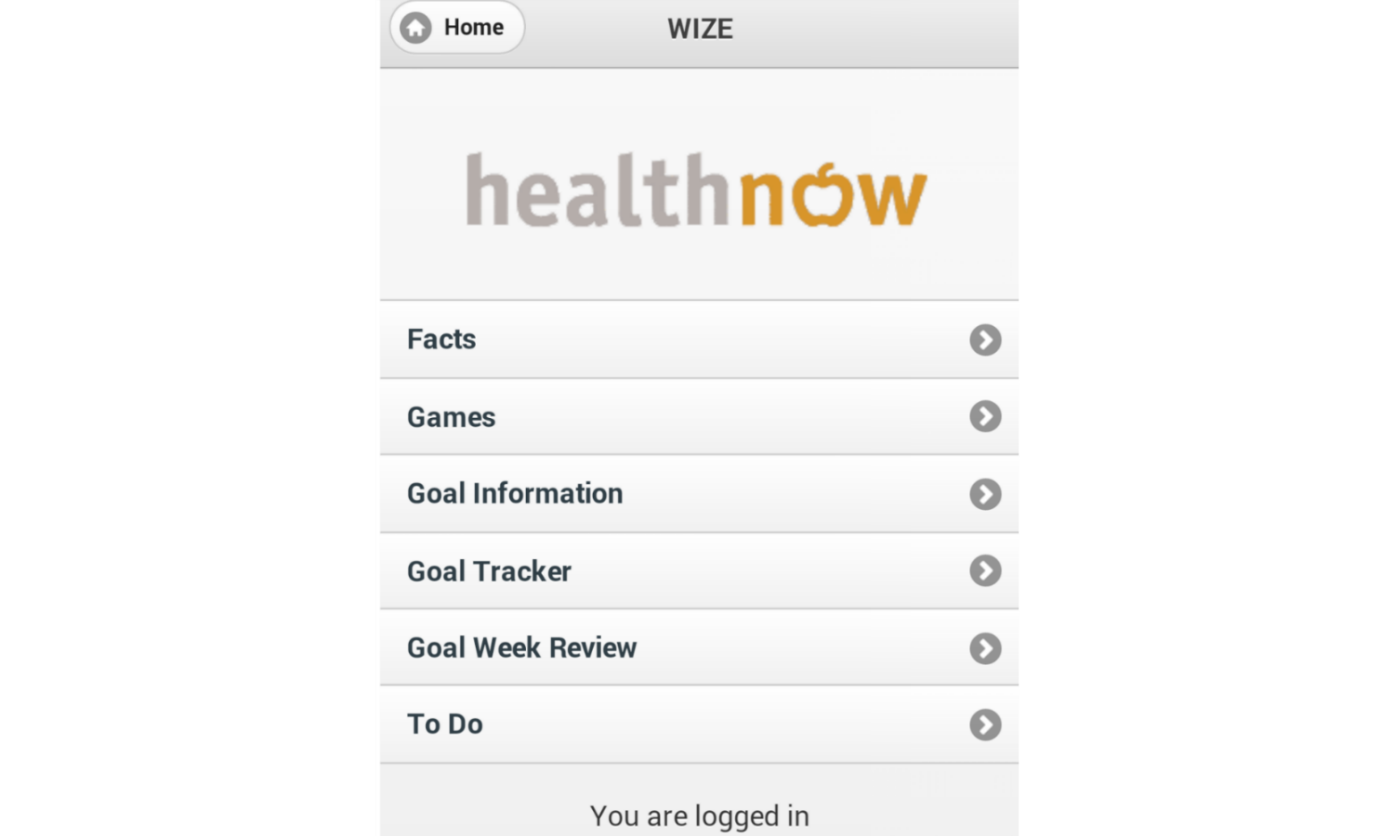
Screenshot of app illustrating home page.

**Figure 2:**
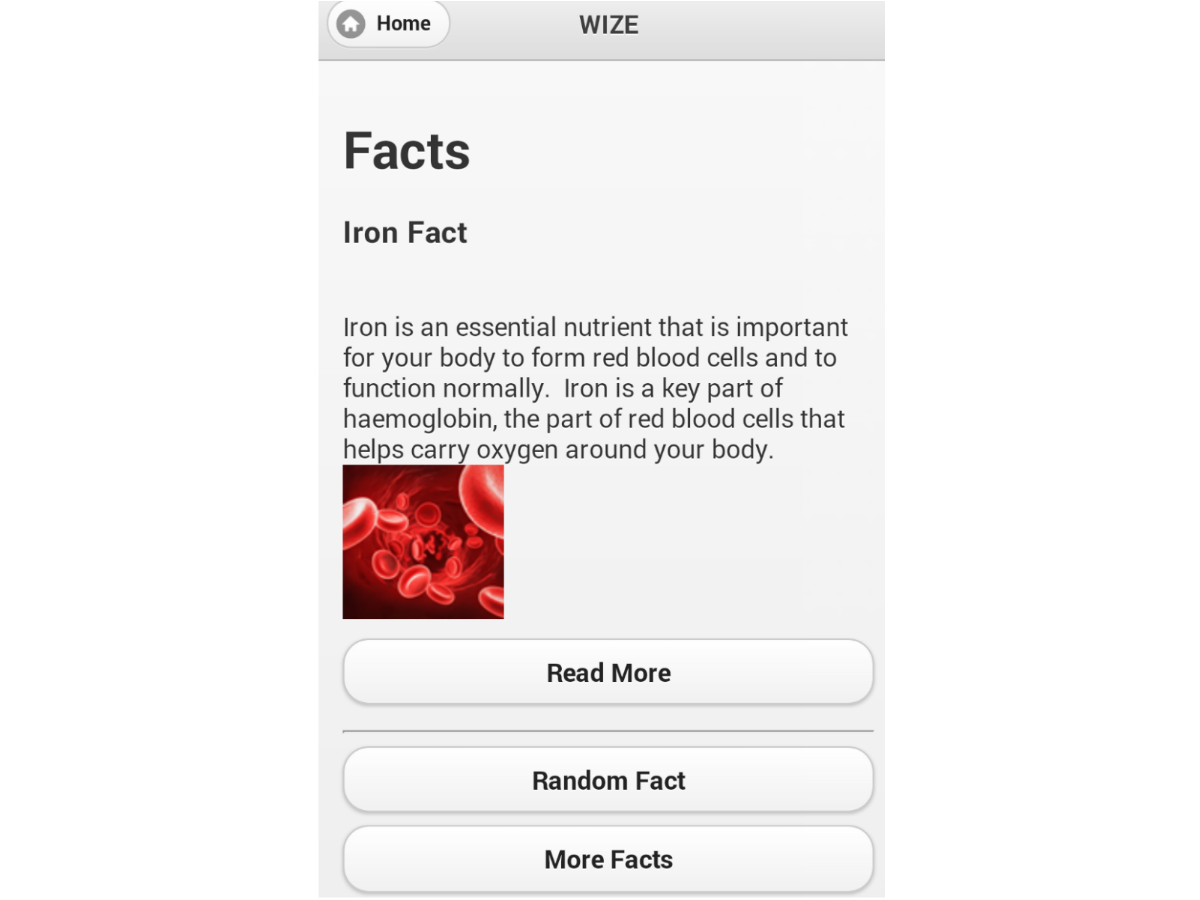
Screenshot of app providing an example of a fact about iron.

**Figure 3:**
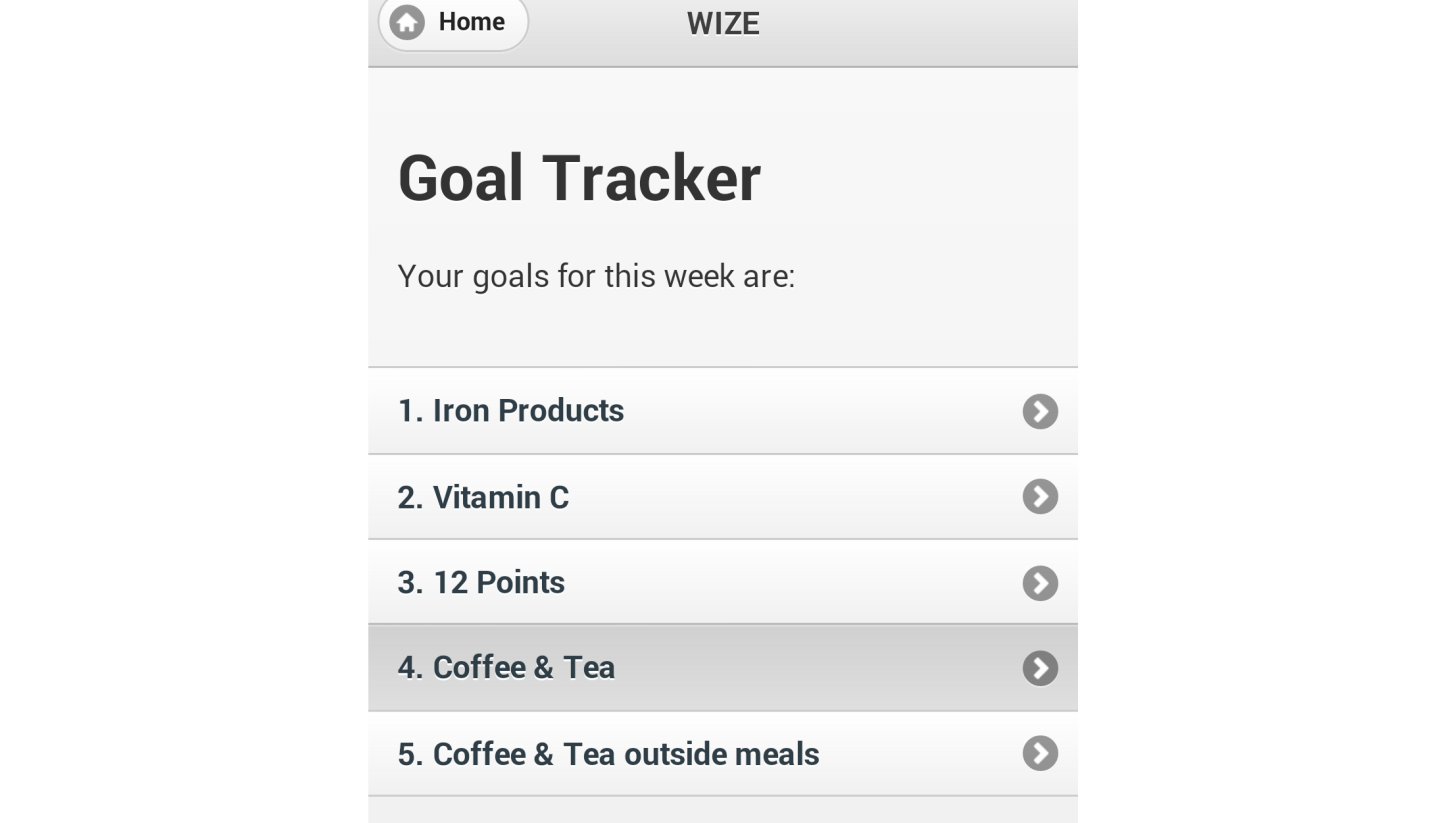
Screenshot of app showing goal tracker function.

The use of games has previously been shown to encourage knowledge uptake; therefore, games (word search and word jumble) were included to develop further iron-related knowledge and facilitate participant engagement and use of the app [27,28]. One goal per week was established (eat 2 iron-fortified products each day, eat 50 mg of vitamin C with meals, aim to eat 12 points of iron each day, and drink coffee and tea at times other than during meals), which aimed to increase iron intake and absorption through gradual implementation of dietary change. To assist women in meeting these goals, a goal tracker was included to record adherence and to monitor participant progress toward each of the goals. The photo diary function was included to help participants monitor and review their food intake if they found they were not meeting specific goals. Screenshots of the app are shown in Figures 1-3.

### Participants

As it is intended that this app be widely available to all premenopausal women interested in increasing intake of bioavailable iron, the current study was open to all women between the ages of 18 and 50 years, irrespective of iron status. However, to use the app, participants were required to have access to an Android mobile phone and be able to communicate in English. Participants were recruited via convenience sampling through flyers placed around Deakin University, Melbourne Burwood Campus, Australia; supermarkets in the surrounding area; free advertisements placed on Facebook pages and Deakin University Student Association club websites; and via presentations given before lectures. All participants filled in a brief background questionnaire collecting information about their age, highest level of education achieved, current degree and major (if studying), current app use (How often do you use apps? What kind of apps do you use? Would you pay for an app?), and current employment status. Ethics approval was granted from Deakin University, Australia and all participants provided written informed consent.

### Focus Groups

After using the app for at least 2 weeks, each participant attended 1 focus group discussion lasting no longer than an hour, and consisting of 6-7 participants per group. Focus group discussions were conducted by 2 researchers with one leading the discussion and the second recording field notes. Verbal data from the focus groups were collected through audio recordings. During the focus group discussion, participants were asked about their likes and dislikes, and reasons for each, for each section of the app. They were also asked about their opinions on the look and feel of the app, content and ease of use, and any suggestions they had to improve the app.

Data collection continued until saturation occurred, which was when no new themes or ideas were emerging from the data [29]. The same researcher (DM) led each focus group.

### Analysis

To help ensure research quality and rigor, approaches highlighted by Braun and Clarke [30] and Fade [31] were employed throughout the study. Audio recordings of the focus groups were transcribed verbatim and anonymized, and a thematic analysis was undertaken [30]. NVivo 10 (QSR International) qualitative research software was used to help manage data and aid analysis [32]. Following familiarization with the data, initial codes were generated in a systematic fashion. Data were collected relevant to each code and themes were then identified and reviewed [30]. An inductive open-coding method was utilized [30]. Codes generated included usability, technological errors, games, facts, and goal tracker. Responses could be coded to more than 1 theme; for example, to both the usability and games themes. Frequencies of code occurrence were then calculated. Samples of analyzed sections of the data were double checked by a second researcher (AOB) to help eliminate bias and ensure accuracy.

## Results

### Participants

Twenty-six participants attended the focus group discussions. The age range of participants was 18-36 years (mean 24.7, SD 5.2). Participants’ iron levels and whether they were pregnant or breastfeeding were unknown. All quotes are presented in intelligent verbatim.

### Themes

Two themes (Table 1) were identified from the focus groups with regard to functionality: technological issues/usability and interface and design. Four themes (Table 1) about the specific components of the app were identified, which included facts, games, goals, and photo diary.

**Table 1:** Themes and their frequency of occurrence (represented in parentheses). Quotes presented in intelligent verbatim.

	Theme	Examples
App component	Facts (78)	*I thought they (facts) were straightforward. I thought they (facts) really seemed to be to the point and provided most of the information I think you needed.* [R3, FG3]
		*There were facts that I didn’t know, which was really cool.* [R3, FG4]
	Games (46)	*Yeah I thought it was cute, a really cool idea cause it’s a health app and health apps don’t really have games. Yeah I really liked it (the games), I thought it was cute.* [R2, FG1]
		*I didn't really get into (the games), I did a couple of the games and things but I did find them very simple and not very useful for me.* [R3, FG3]
		*Yeah a bit more interactive actually was kind of learning.* [R4, FG4]
		*Yeah no real sense of achievement for knowing what a word was.* [R3, FG1]
	Goals (121)	*I wasn’t sure what that meant (in reference to a goal about coffee and tea) and it didn't give much advice. This is the suggested strategy or step that you can take.* [R6, FG3]
		*Yeah I don't think it is so much the goal itself is unachievable but the understanding and facilitating (of the goal) could be hard for sure.* [R5, FG2]
		*...you just want a reminder.* [R3, FG4]
	Photo Diary (40)	*What would you get out of it (photo diary) though?* [R1, FG1]
		*Yeah you can hardly tell by a photo though.* [R3, FG1]
Functionality	Interface and design (including pictures) (134)	*I liked the plain interface on the first page but I think more images throughout would have made it a little bit more exciting.* [R2, FG3]
		*I kind of felt like you were entering it in when you did something for the day, then you had to enter it (completed goals) again when you achieved the goal. Can’t they (goals and goal tracker) just be linked? I just feel like they (goals and goal tracker) should link.* [R3, FG1]
	Usability (86)	*Mine (app) got a bit slow sometimes.* [R4, FG2]
		*Well I had some issues when logging in, at first it took me about ten attempts to log in, but now it’s kept me logged in and that’s fine.* [R2, FG2]

###  App Components

#### Facts

Most participants thought that the facts were succinct, interesting, easy to understand, informative, and were considered by many participants as the best component of the app. Participants wanted more facts and the possibility of an extended information section if they were particularly interested in a specific fact.

Maybe a bit more points (in the facts). Not just one thing about iron, maybe a few things. Just a little bit more information.

R7, FG1

####  Games

Participants’ opinions of the games were mixed. Many participants suggested that the games could provide further education; for example, through a quiz. It was believed this would help to reinforce information provided within the facts and increase the knowledge acquired by participants. In addition, participants believed that an incentive should have been provided to help them achieve a sense of accomplishment once a game was finished or after a correct answer.

#### Goals

Users considered the goals unclear; participants would have liked more details and further clarification of the goals, which was not provided. Participants did not know how to go about achieving these goals and were frustrated that the app did not explain what the goals were or how to achieve them.

I feel there wasn't maybe enough information (in the goals) about what it was that we needed to answer the question, so it was 12 points of iron and I don't know what that means.

R4, FG1

Many participants believed that having prompts or *little pop-up reminders* for the goals would help them remember to complete and enter their goals into the app. One suggestion from participants was to have the weekly goal be an overview of the daily goals instead of having to reenter the details of the completed daily goals into the weekly goal section. Alternatively, instead of having a weekly goal, participants suggested providing an overview of progress in achieving goals and feedback or strategies to help achieve goals.

Being able to upload to tell it what you have eaten and stuff and having it tell you if you have achieved your goals or not, and it would give you a message confirming that you have achieved your goal for that week or that day, a little bit more satisfaction, congratulating you almost.

R4, FG1

####  Photo Diary

Many participants were unreceptive to the notion of using the photo diary component of the app, as they questioned the intent and function of this component. Participants did not believe the photo diary function would be helpful in increasing consumption of high-iron products or meals and did not understand how it could help in facilitating them to achieve their goals.

What was the actual intent of the photo diary?

R2, FG1

###  Functionality

Overall, participants enjoyed the concept of being able to use an app to increase bioavailable iron.

I definitely would (use the app) cause it's not something I would normally keep track of myself so having an app it’s ... cause I am pretty bad with all that kind of health and all that sort of stuff so I liked it, but it needs to be refined.

R4, FG2

However, due to user interface and design issues, they thought the current app was too cumbersome to use, and would not use it themselves.

####  Interface and Design

Participants described the interface as simple and basic. This was stated as the preferred interface design for an app of this platform. Participants thought it would be good to be able to personalize or tailor the app to the individual.

I think the actual icon for the app was a little bit misleading. It was called WIZE, I was just looking for it this morning, and I was like, what is this.

R3, FG2

A hindrance and disliked aspect was the difficulty in navigating through the app (eg, no back button, *clunkiness,* and the inability of participants to edit their inputted daily goals) as well as a lack of color and visuals within the app, giving it a *clinical* appearance. Some participants described the games as a *drag* that did not *entice* the user to keep playing them or using the app. Most participants believed the addition of more pictures, colors, optional noises, and visuals would help to *jazz it up (the interface).*

####  Usability

Participants believed there was decreased usability of the app due to the lack of prompts and reminders, which would enable them to remember to use the app and track goals. Additionally, providing feedback or reviews of user goal progression were considered necessary components to ensure that participants would be motivated to continue to use the app.

You’re not getting the feedback (of how you are progressing) so I was yeah.

FG2, R2

###  Suggestions

Numerous suggestions were mentioned during the focus group discussions, including the addition of a back button, and inclusion of more colors and pictures to help decrease *clunkiness* and improve the interface and design of the app. Including more facts, additional games, and clarification of dietary goals are examples of suggestions previously mentioned.

## Discussion

### Principal Findings

The purpose of this study was to undertake a formative evaluation and identify user preferences for an app designed to improve intake of dietary iron, with the aim to aid app development and ultimately to enhance app uptake and efficacy. This information can also be used to assist in the development of a range of mobile health (mHealth) apps targeting individuals within the studied demographic (women aged 18-50) to undertake dietary change. Overall, participants liked the concept of an app focused on iron and commented that they would find it useful to help track their iron intake. This reinforces the notion that there is a market for an iron-specific app and that the target audience for the app (a population at-risk for ID) have a desire to track and improve their iron status via dietary interventions. Previous studies have shown that dietary and lifestyle interventions utilizing the app platform have also been met with similar positive reception [33-37]. These results suggest that the use of apps for health is accepted by the general population and users have a desire for uptake.

###  Knowledge

Participants wanted more facts and information, as well as the option to have an additional information section, highlighting that the majority of participants did have a desire for knowledge about iron and health. This desire for knowledge is positive, as it has been shown that knowledge is one of many influences that can affect eating behavior [38]. However, knowledge will fail to be effective when not combined with other behavior change strategies [39]. This was discussed during the focus groups, as many participants found the information was not sufficiently connected with easily understood goals or dietary recommendations.

Games have the potential to increase user knowledge, user interactivity, and engagement with the app, while maintaining engagement [27,28]. Thus, to help further knowledge acquisition while maintaining engagement, an information-seeking platform could be integrated into the games.

###  Feedback, Prompts, and Reminders

Users enjoyed the features of monitoring and tracking behavior, setting goals, and the ability to review progress. Many users stated that feedback was an important component that they would like to see incorporated into the app. This is consistent with the findings of similar research that young adults like monitoring and tracking features [40-42]. Feedback has been highlighted as an integral part of successful behavior change and should be incorporated into interventions delivered through apps [43].

Participants described that they wanted prompts as a reminder to use the app and complete goals. This is consistent with the literature, which indicates that prompts can be considered helpful and useful, may increase motivation, and may remind users of their goals [42,44]. However, reminders and prompts have also been considered annoying, as reminders that are perceived to be too frequent may be viewed as unnecessary [40,42]. Differences in the perceived value of reminders may be due to differences in frequency of use, as reminders considered to be unobtrusive or minimally disruptive have been positively received in other studies [45]. It is important that the user has the ability to tailor the frequency of reminders to ensure that they do not become intrusive and are found to be helpful [42].

Participants mentioned that they would like the app to be personalized and tailored to the individual. The ability to tailor the frequency of messages and the content of feedback has been a recurring theme among participants in previous studies. This research has indicated that a health app should be tailored to ensure it is flexible enough to adapt to a user’s lifestyle [33,42]. It has been noted that instant feedback may place too much pressure on users and may frustrate them if they are not achieving their goals [46]. There is also evidence that interventions with individually tailored material have greater retention rates and result in participants spending more time engaged with the interventions [44,47]. Therefore, it is recommended that tailoring or personalization be incorporated into the app during redevelopment.

### Self-Monitoring and Goals

Many users did not understand a few of the goals, emphasizing that goals should not assume user knowledge and should ideally be simple and explained in significant detail to ensure that they are understood and are achievable by the user. Goals that are considered general, which do not provide feedback to notify users on goal progression, have been shown to be less effective than goals that are specific [48].

Many participants suggested that they would have liked the weekly goal review to be an overview or report about progress toward their goals, ideally in graphical form. This finding is not unique; participants in other health-related research on weight and diabetes management have similarly found monitoring to be valuable [49,50]. It has also been found that app users prefer graphical feedback to feedback in other formats [42]. Thus, it may be beneficial for feedback and monitoring in the app to be displayed in a graphical format, which is possible through the mobile phone app platform [42].

Participants stated that rewards would encourage engagement with the app and adherence to dietary goals. Rewards that recognize an achievement may help to motivate participants to continue to use the app and help to maintain user engagement [44,51].

###  Functionality

####  Interface and Design

Mobile app simplicity has been highlighted as one of the most important features for health intervention apps. Simplicity, as indicated by a simple user-friendly interface, has been shown to encourage continued use of health apps [19,33]. This study reinforced this notion as the simple interface was considered a positive attribute of the app. The importance of an attractive user interface has been emphasized in previous studies and was demonstrated in this study, as many participants stated that the lack of color or visuals within the app did not entice them to or discouraged them from using it [41,44]. Therefore, to help engage users the addition of pictures and colors is recommended.

In addition, it appears that personalization or customization of the app was important to participants as it was brought up numerous times. Customization may increase the interactivity that participants have with the app and allow the app to be tailored to an individual [19].

####  Usability

The longer someone engages with an intervention, the more likely the intervention is to be effective [52]. To ensure engagement time, it is recommended that interventions be interactive, tailored, and relevant to participants [52,53]. Discontinued use of mHealth has been attributed to lack of usability and perceived usefulness of the intervention [54,55]. Participants wanting to discontinue using our app due to usability and functionality issues (eg, no back button, *clunkiness,* and the inability to edit inputted daily goals) reinforces the notion that usability is a key component of mHealth uptake. In addition, it has been shown that users must find apps usable and useful to engage in long-term use; thus, it is important that the platform is engaging and entices participants to spend time using the actual intervention [52,56].

### Implementation of Modifications

**Figure 4:**
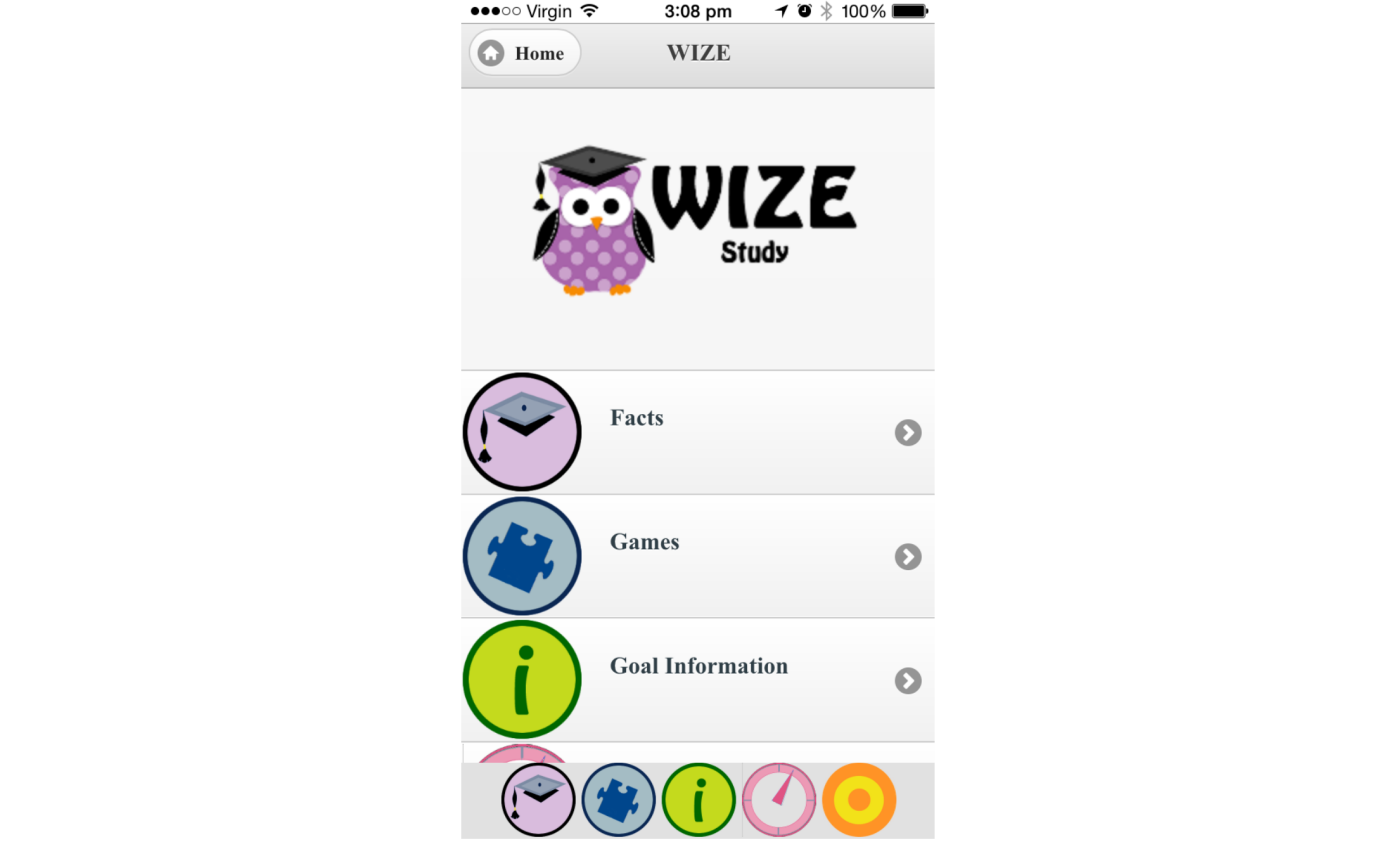
Screenshot of app illustrating home page postmodification.

**Figure 5:**
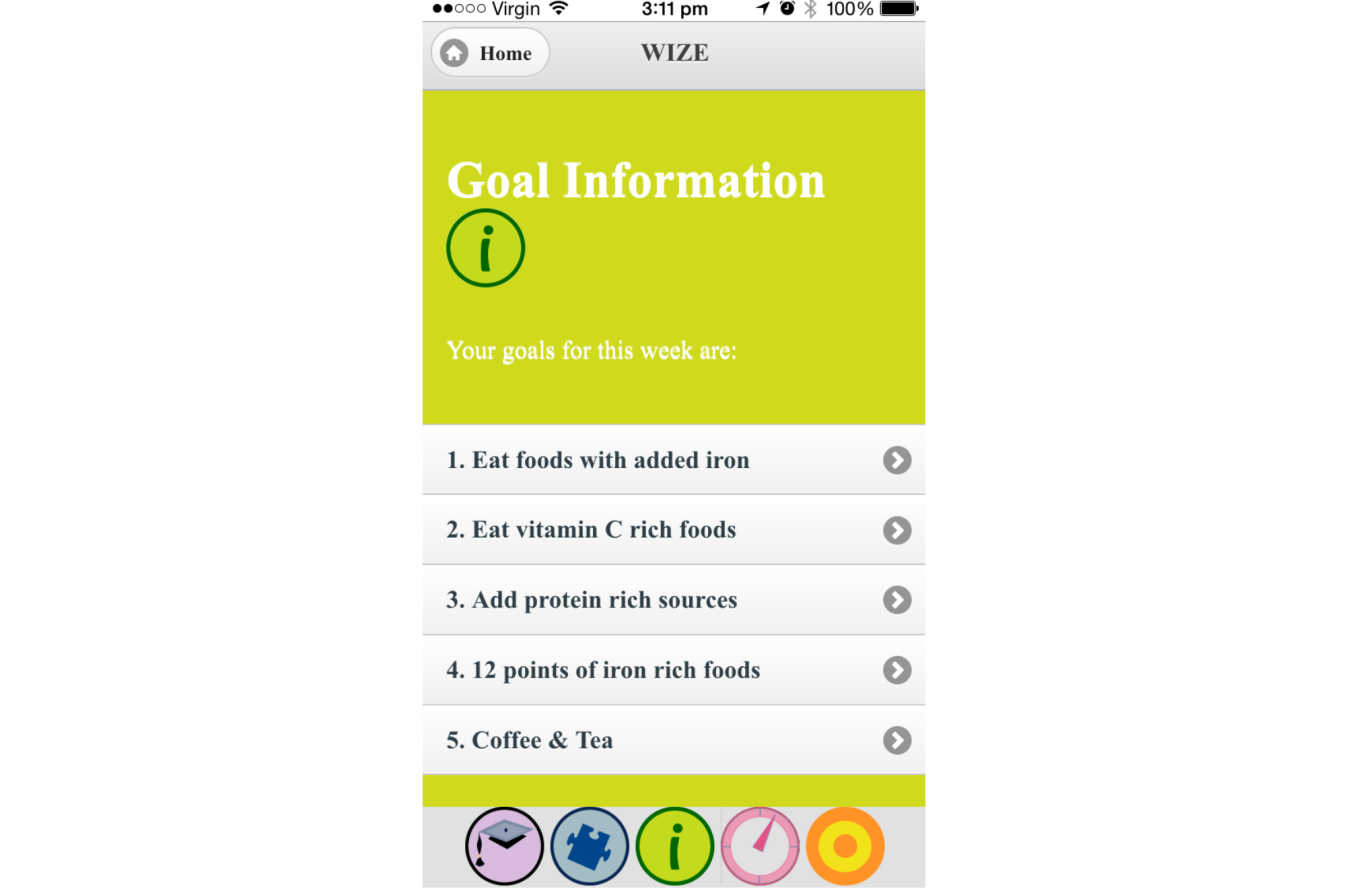
Screenshot of app showing goal tracker function postmodification.

Modifications based on the suggestions from participants in this study were made to the WIZE app. Additional colors and pictures were added throughout the app to increase its appeal; additional links between components of the app were added to increase usability; the goals were clarified and restructured; and games were modified to include further information about iron. Postmodification screen shots of the app can be seen in Figures 4 and 5.

###  Limitations

The majority of participants were recruited from one area of Deakin University, Melbourne Burwood Campus, Australia; therefore, it is unknown if these views are reflective of the broader population. The sample was predominately younger females with a high level of education. Participants having access for only 2 weeks before participating in a focus group may have resulted in different opinions than if the app had been used over a 4-week trial (the period over which goals were designed to be implemented). A further potential limitation was that the app was only available on the Android mobile phone platform. Users of this specific platform may not represent all mobile phone users.

### Conclusion

Participants expressed interest in an app to increase their intake of bioavailable iron and data obtained aided the redevelopment of the app. In terms of components, goals, feedback, and self-monitoring were considered valuable and should be incorporated into future app interventions. The value of implementing other features such as reminders and prompts is unclear and may depend on the individual. Information and insights obtained from this study help to clarify the acceptability and user preferences of apps as a platform for behavior change interventions and may help guide and inform the development of future app interventions.

## Abbreviations

ID: iron deficiency
mHealth: mobile health
